# Erythema Ab Igne from Heating Pad Use: A Report of Three Clinical Cases and a Differential Diagnosis

**DOI:** 10.7759/cureus.2635

**Published:** 2018-05-16

**Authors:** Alexander B Aria, Leon Chen, Sirunya Silapunt

**Affiliations:** 1 Dermatology, University of Texas Mcgovern Medical School at Houston, Houston, USA

**Keywords:** erythema ab igne, heating pad

## Abstract

Erythema ab igne is an asymptomatic cutaneous disorder characterized by erythematous reticulated hyperpigmentation resulting from chronic exposure to infrared radiation in the form of heat. We report three cases of erythema ab igne from chronic heating pad use over a duration of six months to three years. The lesions were asymptomatic in all three patients and were incidental skin findings in two patients, unrelated to their chief complaints. This illustrates the importance of recognizing the morphology and distribution of erythema ab igne. Additionally, knowledge of similarly presenting cutaneous diseases is important to distinguish erythema ab igne from other more worrisome entities that would require further evaluation. Our patients were informed of the benign nature of this condition and were told that cessation of heating pad use would likely result in the resolution of their lesions.

## Introduction

Chronic exposure to heat and infrared radiation may result in reticulated erythematous and hyperpigmented skin lesions known as erythema ab igne. This benign condition is asymptomatic and is traditionally associated with stoves or open fires. The hyperpigmentation of erythema ab igne occurs in relation to heat exposure and usually resolves with the cessation of this exposure; however, permanent hyperpigmentation can occur. We report three cases of erythema ab igne from chronic heating pad use.

## Case presentation

Patient 1

**Figure 1:**
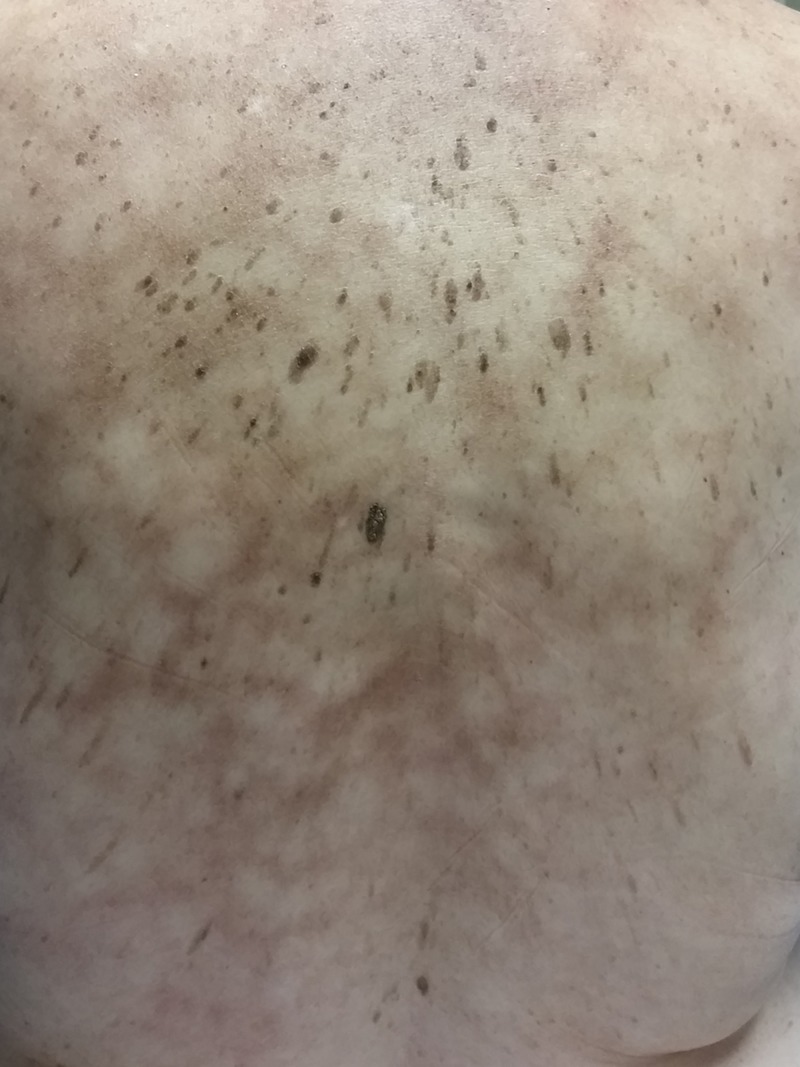
Patient 1. Erythema ab igne Erythematous reticulated hyperpigmentation on the back.

Our first patient was a 76-year-old Caucasian female with skin color changes on her back which were noticed by her husband a few weeks prior to presentation. The patient had a history of generalized pain with no identifiable etiology and had been using an electrical heating pad for 12 months for pain alleviation. As she became bedridden due to her intractable pain, she often laid on the electrical heating pad for at least six consecutive hours for several months and denied any associated burning or discomfort. Physical examination revealed reticulated, ill-defined, reddish-brown patches in a cape-like distribution down the patient’s back (Figure 1).

The patient was informed that her lesions were due to chronic heat exposure and was advised to discontinue using her heating pad. At a follow-up visit 18 months later, her lesions had resolved.

Patient 2

**Figure 2:**
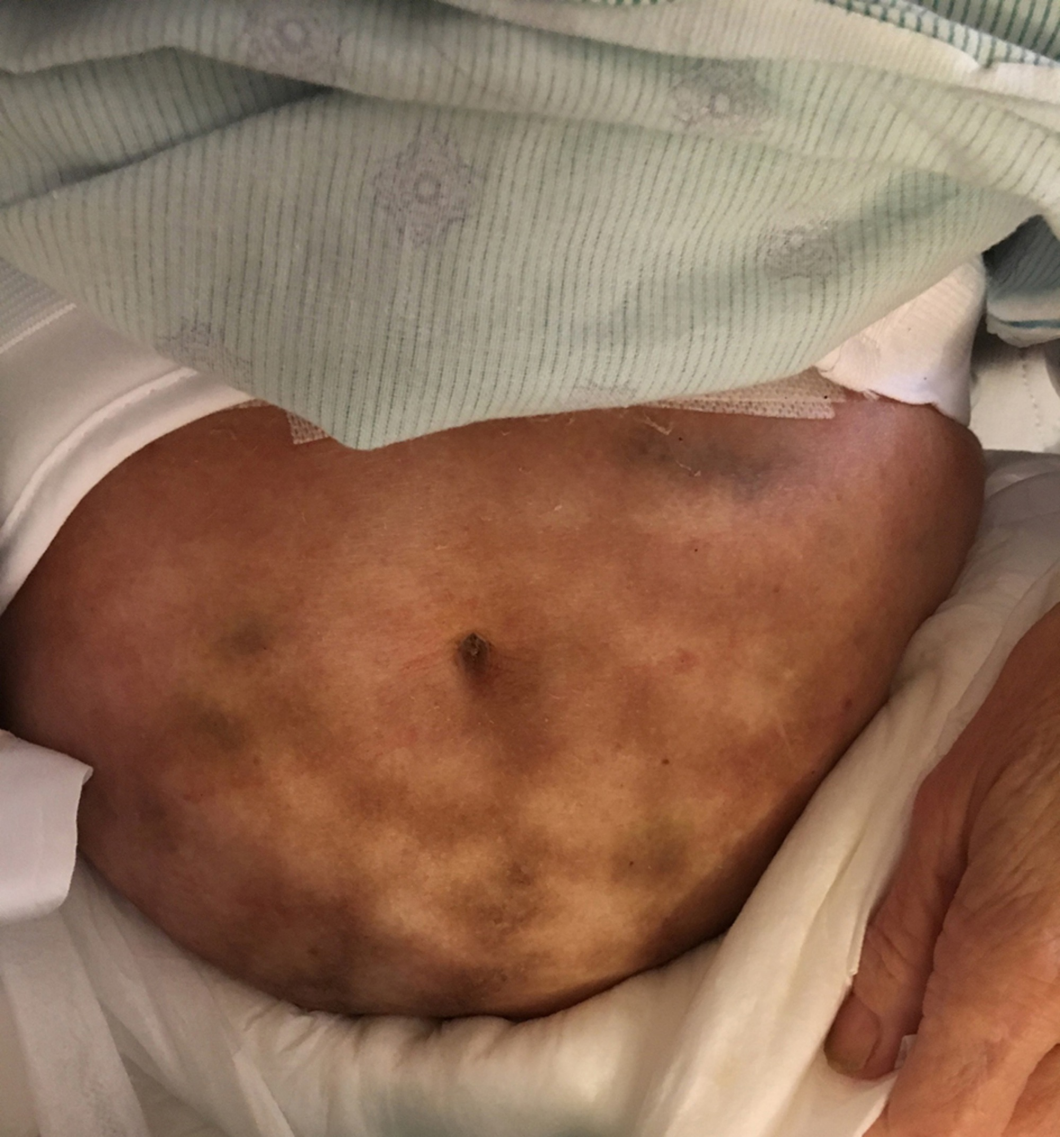
Patient 2: Erythema ab igne Reddish-brown reticulated patches on the lower abdomen.

Our second patient was a 52-year-old Caucasian female seen as an inpatient consult due to the presence of hyperpigmented lesions on her abdomen and upper thighs. She had been admitted to the hospital due to a brain abscess and had undergone a craniotomy for abscess drainage. The patient’s mental status was impaired and a history could not be taken. The primary team stated that the patient’s skin lesions had been present since admission; however, their exact duration was unknown. On examination, the patient had lace-like hyperpigmented patches on the lower abdomen and upper thighs (Figure 2).

The diagnosis of erythema ab igne was given, and the care team was reassured of the benign nature of this condition. Several days later, additional history was obtained from the patient’s husband when he became available. He reported that the patient had uterine fibroids and had been using a heating pad for more than eight hours daily for the past three years to alleviate lower abdominal pain. The husband was informed that the heating pad was the culprit of the patient’s hyperpigmented skin and was instructed to discontinue its use.

Patient 3

**Figure 3:**
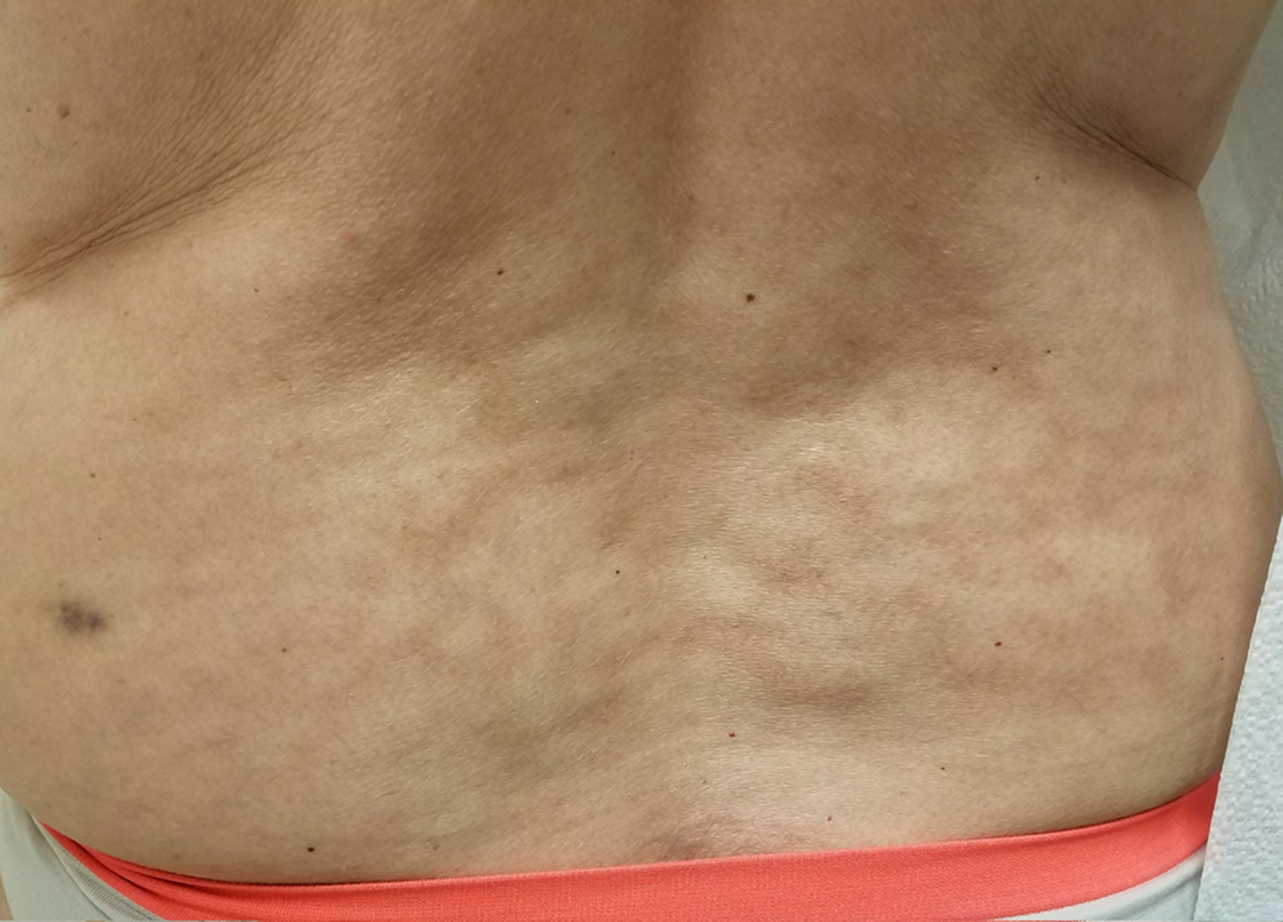
Patient 3: Erythema ab igne Reticulated hyperpigmented patches on the lower back.

Our third patient was a 50-year-old Caucasian female seen in our clinic for a full body skin examination. Hyperpigmented reticulated patches on the lower back were noted incidentally during her physical examination (Figure 3).

The patient was not aware of the lesions but reported that she had used a heating pad weekly for lower back pain for at least six months until the pain resolved a few months prior to her visit. The patient was informed that she had erythema ab igne due to chronic heat exposure and was counseled on its benign course.

## Discussion

In modern society, chronic exposure to heat from laptops [1] or heating pads [2] has become the most common cause of erythema ab igne. Patients will often overlook these skin lesions due to their symptomless nature. Our three cases demonstrate the importance of recognizing morphology and distribution when diagnosing this cutaneous condition, especially in the case of our second patient who was unable to provide any history due to impaired mental status. Knowledge of similarly appearing cutaneous diseases is important for determining whether the underlying cause of a patient’s erythematous hyperpigmented lesions is benign, such as erythema ab igne, or a more worrisome entity that would require further work up.

Other conditions with a similar reticulated appearance as erythema ab igne include livedo reticularis, livedo racemosa, cutis marmorata, and cutis marmorata telangiectatica congenita (Table 1) [3].

**Table 1:** Erythema ab igne and its clinical mimickers APS – antiphospholipid syndrome; SLE – systemic lupus erythematous

Diagnosis	Population affected	Clinical morphology	Distribution	Blanchable	Associations	Treatment
Erythema ab igne	Most frequently middle-aged or older women; recent reports of young adults	Localized reticulated erythema that correlates with a vascular pattern; becomes increasingly hyperpigmented with time	Skin surface exposed to heating source	Early – yes; late – no	Due to chronic heat exposure; development of squamous cell carcinoma or Merkel cell carcinoma has been reported	Removal of heat source or decreasing the exposure duration; can try 5-fluorouracil^1^
Livedo reticularis	Young to middle-aged women	Mottled, net-like pattern of hyperpigmentation that is uniform, symmetric, and reversible	Primarily on the extremities	Yes	Can be idiopathic; due to an underlying disease (APS); physiologic	Treat underlying cause
Livedo racemosa	Young to middle-aged women	Mottled, net-like pattern of hyperpigmentation that is permanent with “irregular” broken netting	Primarily the proximal limbs and trunk	Partially	Can be associated with Sneddon syndrome, SLE, APS, etc.	Treat underlying cause
Cutis marmorata	Neonates, infants	Fluctuant, mottled, net-like pattern of hyperpigmentation	Primarily the lower extremities	Yes	Exposure to cold temperature	Rewarming
Cutis marmorata telangiectatica congenita	Neonates	Reticulated vascular pattern that is persistent	Primarily limited to a single extremity; if trunk is involved, there is usually sharp demarcation at the midline	Partially	Congenital disorder associated with limb asymmetry and vascular malformations as well as neurologic or ocular abnormalities	Usually improves with time

Livedo reticularis is a benign disorder that most often affects young females; can be physiologic, primary, or idiopathic; and is characterized by a persistent or transient reticulated cyanotic pattern [4]. Physiologic livedo reticularis is also known as cutis marmorata [4]. It commonly occurs after exposure to cold temperatures and slowly resolves with rewarming. Unlike livedo reticularis, livedo racemosa is associated with a number of pathological conditions such as systemic lupus erythematosus, antiphospholipid syndrome, and Sneddon syndrome, a non-vasculitic condition presenting with reticulated hyperpigmented lesions and cerebrovascular disease [3-4]. Livedo racemosa is characterized by a violaceous broken reticulated pattern that appears more generalized, widespread, and irregular in shape than livedo reticularis [5]. Cutis marmorata telangiectatica congenita is a congenital disorder which appears as reticulated erythema that usually improves with time and is sometimes associated with limb asymmetry and vascular malformations as well as neurologic or ocular abnormalities [6].

Although erythema ab igne is primarily a clinical diagnosis, histopathology may aid in confirming the diagnosis. Histopathology often demonstrates epidermal keratinocyte atypia, elastosis in the dermis and occasionally liquefaction degeneration of the basal layer, as well as melanin incontinence and hemosiderin in the dermis. Staining with elastic stain Verhoeff-Van Gieson stain can also support the diagnosis of erythema ab igne [7-8].

When evaluating a patient with reticulated hyperpigmented erythema, the diagnosis of erythema ab igne should be considered if there is a history of chronic heat exposure with a corresponding distribution. Malignancy workup may be necessary if there is a high index of suspicion that the heating pad is being used to alleviate pain due to underlying primary malignancy, metastatic disease, or chronic pancreatitis [9]. Although erythema ab igne is typically benign and will likely resolve with discontinuation of heat exposure, secondary development of cutaneous malignancies such as squamous cell carcinomas and Merkel cell carcinomas within the affected area have been reported in the literature [10-11]. If a non-healing wound or ulceration is noted within an erythema ab igne lesion, a skin biopsy is warranted to rule out malignancy. There is no precise data on the duration of heating pad usage required to develop erythema ab igne; however, it is generally recommended that the treatment time should not exceed 30 minutes [12].

## Conclusions

The above three cases illustrate the importance of recognizing the morphology and distribution of erythema ab igne apart from other clinical mimickers. Complete cessation of heating pad use typically results in resolution of these erythema ab igne lesions.
